# *In vivo* quantification of embryonic and placental growth during gestation in mice using micro-ultrasound

**DOI:** 10.1186/1477-7827-6-34

**Published:** 2008-08-12

**Authors:** Junwu Mu, John C Slevin, Dawei Qu, Sarah McCormick, S Lee Adamson

**Affiliations:** 1 Samuel Lunenfeld Research Institute of Mount Sinai Hospital, Toronto, Canada; 2 Department of Obstetrics & Gynecology, University of Toronto, Toronto, Canada; 3 Department of Physiology, University of Toronto, Toronto, Canada; 4 Department of Medical Biophysics, University of Toronto, Toronto, Canada

## Abstract

**Background:**

Non-invasive micro-ultrasound was evaluated as a method to quantify intrauterine growth phenotypes in mice. Improved methods are required to accelerate research using genetically-altered mice to investigate the interactive roles of genes and environments on embryonic and placental growth. We determined (1) feasible age ranges for measuring specific variables, (2) normative growth curves, (3) accuracy of ultrasound measurements in comparison with light microscopy, and (4) weight prediction equations using regression analysis for CD-1 mice and evaluated their accuracy when applied to other mouse strains.

**Methods:**

We used 30–40 MHz ultrasound to quantify embryonic and placental morphometry in isoflurane-anesthetized pregnant CD-1 mice from embryonic day 7.5 (E7.5) to E18.5 (full-term), and for C57Bl/6J, B6CBAF1, and hIGFBP1 pregnant transgenic mice at E17.5.

**Results:**

Gestational sac dimension provided the earliest measure of conceptus size. Sac dimension derived using regression analysis increased from 0.84 mm at E7.5 to 6.44 mm at E11.5 when it was discontinued. The earliest measurement of embryo size was crown-rump length (CRL) which increased from 1.88 mm at E8.5 to 16.22 mm at E16.5 after which it exceeded the field of view. From E10.5 to E18.5 (full term), progressive increases were observed in embryonic biparietal diameter (BPD) (0.79 mm to 7.55 mm at E18.5), abdominal circumference (AC) (4.91 mm to 26.56 mm), and eye lens diameter (0.20 mm to 0.93 mm). Ossified femur length was measureable from E15.5 (1.06 mm) and increased linearly to 2.23 mm at E18.5. In contrast, placental diameter (PD) and placental thickness (PT) increased from E10.5 to E14.5 then remained constant to term in accord with placental weight. Ultrasound and light microscopy measurements agreed with no significant bias and a discrepancy of less than 25%. Regression equations predicting gestational age from individual variables, and embryonic weight (BW) from CRL, BPD, and AC were obtained. The prediction equation BW = -0.757 + 0.0453 (CRL) + 0.0334 (AC) derived from CD-1 data predicted embryonic weights at E17.5 in three other strains of mice with a mean discrepancy of less than 16%.

**Conclusion:**

Micro-ultrasound provides a feasible tool for in vivo morphometric quantification of embryonic and placental growth parameters in mice and for estimation of embryonic gestational age and/or body weight in utero.

## Background

Genetically-altered mouse models are proving powerful tools for studying the genetic regulation of embryonic and placental growth and development [1-3], and the interaction between genes and the environment on intrauterine and postnatal growth [4]. Advancing knowledge gained from such models is important given the critical importance of intrauterine growth as a risk factor for perinatal and childhood morbitity and mortality [5] and for diverse adult-onset diseases including diabetes, cancer, and hypertension [6-8]. Factors regulating intrauterine growth are known to differ from those important postnatally, and they remain poorly understood [9,10]. Thus, methods to monitor embryonic and placental growth efficiently and accurately in utero in mice would accelerate progress in this important area.

Body weight is the most common parameter used to quantify growth but it provides no information on whether growth is proportionate or preferentially affects length, girth, or other body proportions. Placental growth is often neglected despite the critical role of this organ in supporting embryonic growth and maternal adaptations to pregnancy. Abnormal placental size is now recognized as an early predictor of poor fetal growth and poor pregnancy outcome in human pregnancy [11]. Furthermore, detection of a decelerating rate of intrauterine growth using ultrasound improves the sensitivity of detection of compromised human fetuses [12] suggesting serial measurements of growth would also be of value when phenotyping mouse models with intrauterine growth abnormalities. Thus there is a pressing need for methods to quantify prenatal growth characteristics as a function of gestation in genetically-altered and/or environmentally-challenged mice.

Most prior work in mice has evaluated prenatal growth using ex vivo embryonic and/or placental weights as measured variables. In human pregnancy, ultrasound is extensively used to quantify fetal and placental growth, and to estimate fetal gestational age and/or body weight based on morphometric measurements. Measurement parameters include gestational sac dimension, crown-rump length, abdominal circumference, biparietal skull diameter, and femur length [13,14]. Recent work in mice showed these parameters can be measured in embryos in utero using 7.5 to 15 MHz ultrasound [15-18] and can be used to generate prediction equations for gestational age [16,17]. However, information on normal growth trajectories for embryonic parameters is limited and there is no information on placental parameters or on measurement accuracy, and no body weight prediction equations exist for using ultrasound measurements of mouse embryos.

There have been major technological advances in small animal imaging [19,20] including the development of micro-ultrasound [21]. A lateral resolution of ~40 μm is achieved using ~40 MHz ultrasound and this represents an approximate 10-fold improvement over more conventional 15 MHz ultrasound [22]. Micro-ultrasound has been used to quantify growth of the lens of the embryonic eye in mouse embryos from E11.5 to term [23] suggesting that this higher resolution instrumentation might permit growth quantification of other parameters in the embryo and placenta to commence earlier in gestation and provide more precise measurements than previously possible.

In the current study, we used 40 MHz ultrasound to image the postimplantation mouse conceptus and determined (1) feasible age range for measuring specific variables using on-screen digital calipers, (2) normative growth curves and gestation prediction equations, (3) accuracy of ultrasound measurements in comparison with light microscopy, and (4) body weight prediction equations using regression analysis for CD-1 mice and their accuracy when applied to other mouse strains.

## Methods

**Figure 1:**
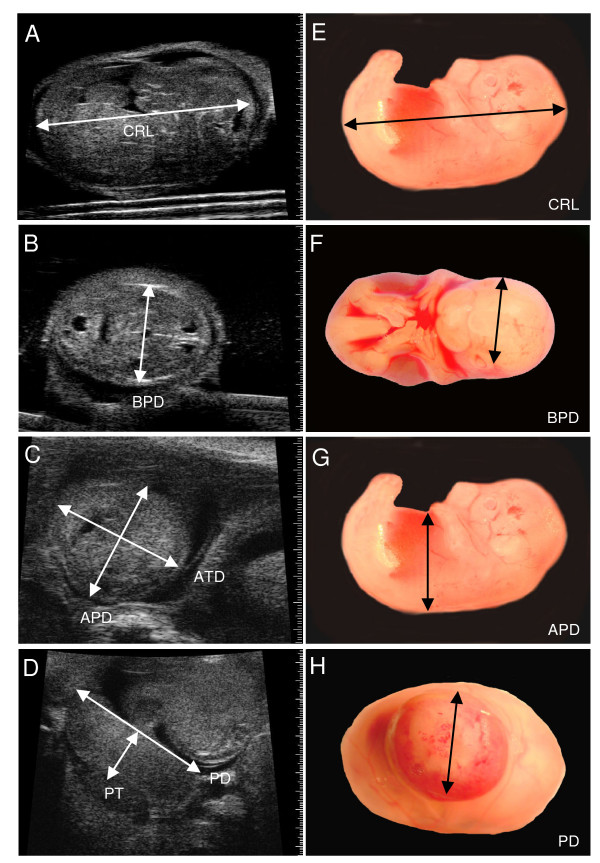
**Validation of ultrasound dimension measurements by light microscopy**. (A-D) Ultrasound images of an embryo at E14.5 illustrating measurement locations for crown-rump length (CRL), biparietal diameter (BPD), abdominal anteroposterior diameter (APD), abdominal transverse diameter (ATD), placental diameter (PD), and placental thickness (PT) and (E-H) obtained ex vivo by light microscopy.

Experiments were approved by the animal care committee of Mount Sinai Hospital and were conducted in accord with guidelines established by the Canadian Council on Animal Care. The normal developmental time-course for growth parameters were obtained in pregnant out-bred mice between 1 and 5 PM (CD-1; Harlan Sprague Dawley, Indianapolis, IN). Mice were on a 12 h light dark cycle, were housed in SPF conditions, and were fed ad-lib (Purina Picolab Rodent Diet 20). Measurements were obtained from transcutaneous, non-invasive ultrasound images obtained from a total of 211 embryos from at least three pregnant mice per gestational day from E7.5 to E18.5. After ultrasound exams, the mouse was killed while still anesthetized, and the embryos and placentas were collected for direct measurement of weight. In some cases, direct measurements of dimensions by light microscopy were made using an eye-piece graticule (Fig. 1) to evaluate the accuracy of the ultrasound measurements. In these cases, the locations of embryos in the abdomen were recorded during the ultrasound exams and the corresponding embryos were identified post mortem (144 embryos from 16 pregnant mice between E11.5 and E18.5 of gestation). These were used for pair-wise comparisons of in utero and ex utero measurements.

Day 0.5 of pregnancy (E0.5) was defined as morning on the day a vaginal plug was found after overnight mating. Mice were lightly anesthetized with ~1.5% isoflurane in oxygen by face mask. Hair was removed from the abdomen by shaving, followed by a chemical hair remover. Pre-warmed gel was used as an ultrasound coupling medium. A 30 MHz or 40 MHz transducer operating at 30 frames/s was used to transcutaneously image embryos within the maternal abdomen (Model Vevo 660, VisualSonics Inc., Toronto, ON, Canada). Maternal heart rate and rectal temperature were monitored (Model THM100; Indus Instruments, Houston, TX), and heating was adjusted to maintain rectal temperature between 36 and 38°C.

**Figure 2:**
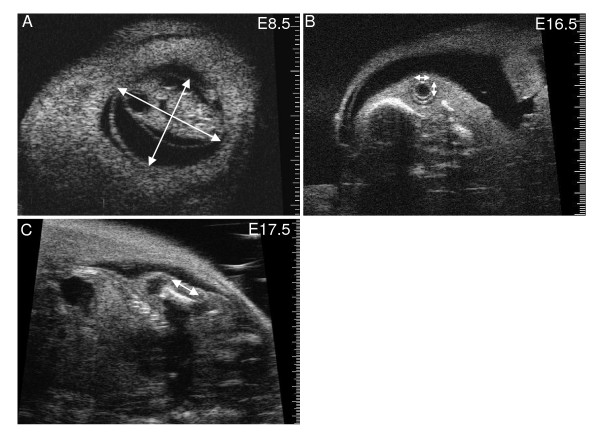
**Ultrasound dimension measurements**. Ultrasound images illustrating where dimension measurements were obtained (arrows). (A) Gestational sac at E8.5. (B) Embryo eye at E16.5 showing lens and surrounding vitreous humor. (C) Longitudinal view of femur at E17.5.

The electronic calipers of the ultrasound software were used to measure embryonic and placental dimensions on the ultrasound screen (Fig. 1, 2). The long axis and the largest dimension perpendicular to the long axis were measured and averaged to provide a measurement of the size of the gestational sac (i.e. the fluid-filled structure containing the embryo which is visualized as an anechoic (dark) space bounded by the surrounding echogenic (white) tissue of the parietal yolk sac) (Fig. 2A). Eye lens diameter was the average of the largest dimension and the orthogonal dimension (Fig. 2B). Femur length was measured on a longitudinal view from the outer edges of the ossified bone (Fig. 2C). The CRL was quantified as the maximum distance from the cephalic pole to the caudal pole (Fig. 1A). The BPD was measured from the outer border of the transverse axial view of the head in which the central midline echo and the lateral ventricles were visible (Fig. 1B). Abdominal circumference (AC) was calculated from the abdominal anteroposterior diameter (APD) and abdominal transverse diameter (ATD) measured from a transverse section of the fetal abdomen at the level of the stomach and the umbilical vein (Fig. 1C), where AC = π (ATD + APD)/2. For placental measurements, a transverse image of the placenta was obtained at the insertion site of the umbilical cord and the placental diameter (PD) was measured. Placental thickness (PT) was measured at the centre of the placenta from the chorionic surface to the echogenic calcium deposits in the giant cell layer [24] (Fig. 1D).

**Figure 3:**
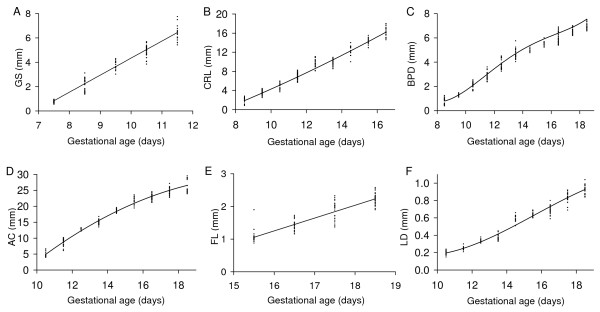
**Embryonic growth quantified using ultrasound parameters**. (A) gestational sac dimension (GS), (B) crown-rump length (CRL), (C) biparietal diameter (BPD), (D) abdominal circumference (AC), (E) femur length (FL), and (F) lens diameter (LD) measured non-invasively by ultrasound in vivo are shown as a function of gestational age. The lines were generated using the regression equation between the parameter and gestational age shown in Table 1. The regression equations were derived using the datapoints shown (each point is the result obtained in one conceptus).

Results are presented as individual embryo values (Fig. 3) or as the values predicted at each gestational age from the regression equations shown in Table 1 and listed in Table 2. A *p *value of < 0.05 was considered statistically significant. Non-linear regression analysis was used to determine the relationship between the parameter and gestational age. Regression analysis was used to generate equations relating fetal weight to measured ultrasound parameters. Agreement between ultrasound and light microscopic measurements was quantified using Bland-Altman analysis [25] and was expressed as the 95% confidence interval for the percent difference (100 × (ultrasound - light microscopy)/average of two methods). Equations derived from CD-1 mice were applied to estimate body weight from ultrasound parameters in embryos from different strains, and the agreement between measured body weight and predicted body weight was expressed as the mean absolute percent discrepancy (100 × (absolute value of predicted - measured weight)/measured weight).

**Table 1:** Prediction equations for growth parameters (in mm) as a function of gestational age (in days)

Gestational sac (GS):
GS = -9.66 + 1.40(GA)
R^2 ^= 0.9602

Crown-rump length (CRL):
CRL = -9.42 + 1.09(GA) + 0.0281(GA)^2^
R^2 ^= 0.9682

Biparietal diameter (BPD):
BPD = 50.47 - 17.14(GA) + 2.07(GA)^2 ^- 0.103(GA)^3 ^+ 0.00186(GA)^4^
R^2 ^= 0.9733

Abdominal circumference (AC):
AC = -55.75 + 7.52(GA) - 0.166(GA)^2^
R^2 ^= 0.9723

Femur length (FL):
FL = -5.02 + 0.392(GA)
R^2 ^= 0.8215

Lens diameter (LD):
LD = 2.205 - 0.518(GA) + 0.0401(GA)^2 ^- 0.000856(GA)^3^
R^2 ^= 0.9618

Body weight (BW):
BW = 0.5488 - 0.01714(GA) - 0.01180(GA)^2 ^+ 0.0008279(GA)^3^
R^2 ^= 0.9906

Placental diameter (PD):
PD = -11.96 + 2.09(GA) - 0.046(GA)^2 ^- 0.0005(GA)^3^
R^2 ^= 0.8941

Placental thickness (PT):
PT = 4.10 - 1.14(GA) + 0.115(GA)^2 ^- 0.0031(GA)^3^
R^2 ^= 0.7562

Placental weight (PW):
PW = -0.54180 + 0.07887(GA) - 0.002243(GA)^2^
R^2 ^= 0.8237

GA, gestational age

**Table 2:** Predicted fetal measurements at each gestational age using regression equations in Table 1

GA (days)	BW (g)	GS (mm)	CRL (mm)	BPD (mm)	AC (mm)	FL (mm)	LD (mm)
7.5		0.84					
8.5		2.24	1.88	0.79			
9.5		3.64	3.47	1.30			
10.5	0.026	5.04	5.12	2.09	4.91		0.20
11.5	0.050	6.44	6.83	3.00	8.78		0.25
12.5	0.108		8.60	3.90	12.31		0.32
13.5	0.204		10.42	4.70	15.52		0.41
14.5	0.343		12.29	5.37	18.39		0.52
15.5	0.531		14.23	5.92	20.93	1.06	0.62
16.5	0.772		16.22	6.39	23.14	1.45	0.73
17.5	1.072			6.89	25.01	1.84	0.83
18.5	1.435			7.55	26.56	2.23	0.93

GA, gestational age, BW, body weight, GS, gestational sac; CRL, crown-rump length; BPD, biparietal diameter; AC, abdominal circumference; FL, femur length; LD, lens diameter

## Results and Discussion

The gestational sac dimension was the earliest quantitative measure of growth and was consistently measurable from E7.5. It provides a measure of the fluid space surrounding the embryo. Gestational sac dimension increased linearly by 1.40 mm/d from 0.84 mm at E7.5 to 6.44 mm at E11.5 when measurement of this parameter was discontinued (Fig. 3A and Table 1, 2). By this method, gestational sac dimension was measurable two days earlier than in prior work using 15.5 MHz ultrasound [16]. Both methods yielded similar gestational sac dimensions at E9.5 (4.4 mm vs. 3.64 mm in the current study).

**Figure 4:**
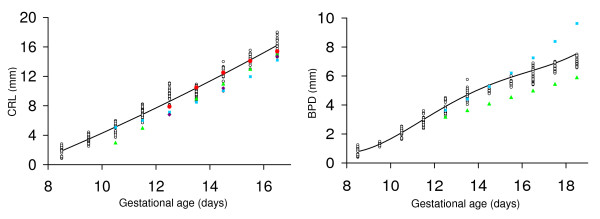
**Comparison of crown-rump length and biparietal diameter measurements with prior work**. Crown-rump length (CRL) and biparietal diameter (BPD) are shown as a function of gestational age. Open symbols show the results obtained for each conceptus in the current study. The solid lines were generated using the regression equations shown in Table 1. Solid symbols show results from prior work. Values were calculated using formulas for CD-1 embryos obtained using 7.5–10 MHz (blue squares; [17]) or 15 MHz (green triangles; [16]) ultrasound or are means reported in Tables for C57Bl/6 embryos (red circles [18], purple diamonds [15]).

**Figure 5:**
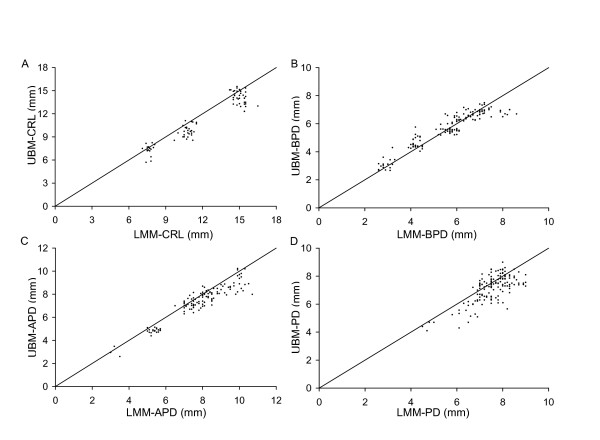
**Correspondence between ultrasound and light microscopy measurements**. Relationship between measurements obtained by ultrasound (UBM) in vivo and by light microscopy (LMM) ex vivo for (A) crown-rump length (CRL), (B) biparietal diameter (BPD), (C) anterioposterior abdominal dimension (APD), and (D) placental diameter (PD). Each point shows the result obtained in one conceptus. The lines show the line of identity (where y = x).

**Figure 6:**
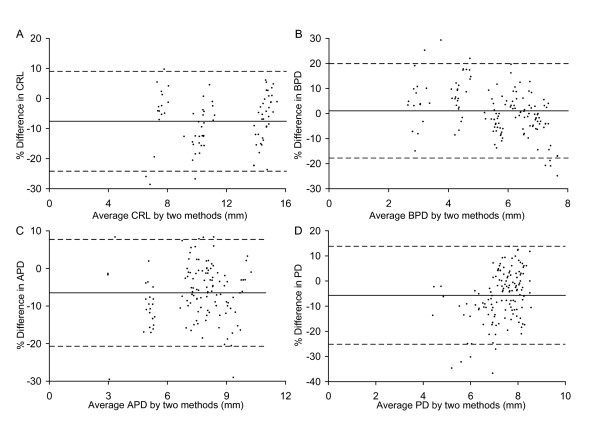
**Statistical evaluation of ultrasound versus light microscopy measurements**. Bland-Altman analyses of the relationship between measurements obtained by ultrasound (UBM) in vivo and by light microscopy (LMM) ex vivo for (A) crown-rump length (CRL), (B) biparietal diameter (BPD), (C) anterioposterior abdominal dimension (APD), and (D) placental diameter (PD). The difference between paired measurements are plotted against the mean of the two measurements. Each point shows the paired results obtained in one conceptus. The solid line in each graph shows the bias between the two measurement methods. The bias was not significantly different from zero for all four variables. The dashed lines show the ± 95% confidence intervals.

The crown-rump length of the embryo was measurable from E8.5 to E16.5 when the length of most CD-1 embryos exceeded the field of view so were no longer measurable. Crown-rump length increased non-linearly from 1.88 mm at E8.5 to 16.22 mm at E16.5 (Fig. 3B and Tables 1, 2). At E8.5 the embryonic headfold is at an early stage of development and the embryo has not yet rotated into the embryonic position characteristic of the rest of gestation. Nevertheless, the 'crown-rump length' measured at this gestation was congruent with the relationship between crown-rump length and gestational age of older embryos (Fig. 3B). Crown-rump length was one of the easiest parameters to measure, and regression analysis showed that it was a good predictor of embryonic body weight and of gestational age (Tables 3, 4). Prior work using 15 MHz ultrasound showed that crown-rump length could be measured as early as E10.5 in CD-1 embryos [16] or E12.5 in C57Bl/6J embryos [15,18]. A more recent publication indicates that crown-rump length is measurable from E5.5 to E18.5 in CD-1 and C57Bl/6J embryos using 7.5–10 MHz ultrasound but the measurement accuracy was not reported at any gestational age. Crown-rump length in the current study tended to be larger than values predicted using formulas for CD-1 embryos [16,17] or reported in Tables for C57Bl/6 embryos [15,18] in prior work using 7.5 to 15 MHz ultrasound (Fig. 4). Nevertheless, we found good agreement between crown-rump length measured by light microscopy ex vivo and ultrasound in vivo (Fig. 5A). Overall, there was no significant bias, and the difference between measurements by the two methods was 25% or less (Fig. 6A). Thus, measurements using lower resolution ultrasound may underestimate crown-rump length.

**Table 3:** Prediction equations for gestational age (in days) from measured variables (in mm)

Gestational sac (GS):
GA (day) = 6.687 + 1.395(GS) - 0.4391(GS)^2 ^+ 0.09837(GS)^3 ^- 0.007091(GS)^4^
R^2 ^= 0.9683

Crown-rump length (CRL):
GA (day) = 7.622 + 0.5264(CRL) + 0.009440(CRL)^2 ^- 0.0005539(CRL)^3^
R^2 ^= 0.9693

Biparietal diameter (BPD):
GA (day) = 8.195 + 0.8689(BPD) + 0.08056(BPD)^2^
R^2 ^= 0.9648

Abdominal circumference (AC):
GA (day) = 7.645 + 0.8774(AC) - 0.07917(AC)^2 ^+ 0.004024(AC)^3 ^- 6.508e^-5^(AC)^4^
R^2 ^= 0.9698

Femur length (FL):
GA (day) = 12.24 + 3.822(FL) - 0.5103(FL)^2^
R^2 ^= 0.8287

Lens diameter (LD):
GA (day) = 11.96 - 84.88(LD) + 1470(LD)^2 ^- 4625(LD)^3^
R^2 ^= 0.7582

GA, gestational age

**Table 4:** Prediction equations for body weight (in g) from measured variables (in mm)

From crown-rump length (CRL):
BW = -0.696 + 0.0890(CRL)
R^2 ^= 0.938

From biparietal diameter (BPD):
BW = -34.08 + 32.10(BPD) - 11.130(BPD)^2 ^+ 1.68(BPD)^3^-0.093(BPD)^4^
R^2 ^= 0.945

From abdominal circumference (AC):
BW = 4.20 - 0.76(AC) + 0.045(AC)^2 ^- 0.00078(AC)^3^
R^2 ^= 0.957

From crown-rump length (CRL) and abdominal circumference (AC):
BW = -0.757 + 0.0453(CRL) + 0.0334(AC)
R^2 ^= 0.962

BW, body weight

Abdominal dimensions were sometimes measurable at E9.5 but were consistently measurable from E10.5 onwards. Abdominal anteroposterior diameter measured by ultrasound in vivo showed good agreement with light microscopic measurement ex vivo (Fig. 5C), with no significant bias and a discrepancy of <21% (Fig. 6C). Abdominal anteroposterior and transverse diameters were used to calculate abdominal circumference. Abdominal circumference provides an indicator of soft tissue growth of abdominal organs, primarily the liver [26,27]. Abdominal circumference increased non-linearly with advancing gestation (Fig. 3D, Table 1, 2). Regression analysis showed that abdominal circumference was a good predictor of embryonic body weight and gestational age (Table 3, 4), which is consistent with prior work in human pregnancy.

Biparietal diameter increased from 0.79 mm at E8.5 (when it was measurable in most embryos) to 7.55 mm at E18.5 based on the non-linear regression equation for biparietal diameter as a function of gestational age (Fig. 3C and Table 1). Biparietal diameter was a good predictor of gestational age (R^2 ^= 0.9648; Table 3) and body weight (R^2 ^= 0.945; Table 4). Biparietal diameters in the current study were generally within the range predicted using formulas published previously for CD-1 embryos (obtained using 7.5 – 10 MHz [17] or 15 MHz ultrasound [16]) (Fig. 4). Whether biparietal diameter was measured by ultrasound in vivo or by a light microscope ex vivo (Fig. 5B), bias was not significant and the difference was <20% (Fig. 6B). Biparietal diameter provides prenatal diagnosis of microcephaly in human pregnancy [28] and may reveal asymmetric growth in intrauterine growth restriction. We note that the bias between ultrasound and light microscopy measurements of biparietal diameter tended to be smaller than for soft tissues (i.e. anterioposterior abdominal dimension, placental diameter, and crown-rump length) (+1% versus -6% to -8%) (Fig. 6). Although biases were not statistically significant, this trend may be caused by the less distinct tissue boundaries for soft tissues when viewed by ultrasound.

The femur was first detectable within the hind limb at ~E15.5 and increased linearly in length to term at a rate of 0.392 mm/d (Fig. 3E and Table 1, 2). Prior work also found that the femur was first visualized by ultrasound at this gestational age and suggested it may be useful as a marker for this stage of development [16]. Ossification of the femur was first detected between E14.5 and E15.5 in ex vivo specimens and was primarily localized to the middle of the femur with the extremities of the femur composed of cartilage [29]. Ultrasound-detectable femur length in the current study is 41% to 46% of that determined ex vivo at E15.5 to E18.5 respectively [29], likely because only the middle, ossified region of the femur is detectable by ultrasound. Nevertheless the growth of the femur determined in the current study over this interval (+110%) is similar to that of the whole femur assessed ex vivo (+88%) [29] suggesting that it is a useful non-invasive measure of long bone growth.

The lens, vitreous humor and retina of the eye were visible by ultrasound from E10.5 onwards (Fig. 2B) as shown previously using similar ultrasound instrumentation and the same mouse strain [23]. Lens diameter increased non-linearly with gestational age from 0.20 mm at E10.5 to 0.93 mm at E18.5 (Fig. 3F, Table 1, 2). We note that non-linearity in our data was primarily due to the earliest age point and thus may reflect slower growth during early differentiation of the eye. When linear regression was applied as in prior work, the linear growth rate was ~90 μm/day which is similar to the 70 μm/day previously reported [23]. Lens diameter increases approximately linearly with gestational age in human fetuses from 15 to 40 weeks gestation [30]. In human fetuses, slow ocular growth is associated with delayed cerebral development [31,32]. Thus a measurement of lens diameter may provide a useful phenotyping marker for eye and, indirectly, brain development in mouse models.

**Figure 7:**
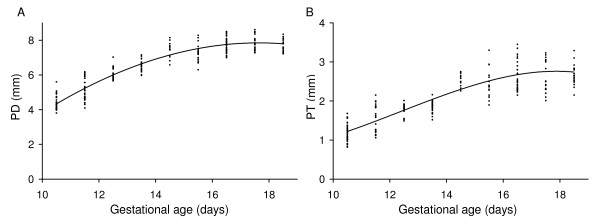
**Placental growth quantified using ultrasound parameters**. Relationship between (A) placental diameter (PD) and (B) placental thickness (PT). The lines were generated using the regression equation between the parameter and gestational age shown in Table 1. The regression equations were derived using the datapoints shown (each point is the result obtained in one conceptus).

**Figure 8:**
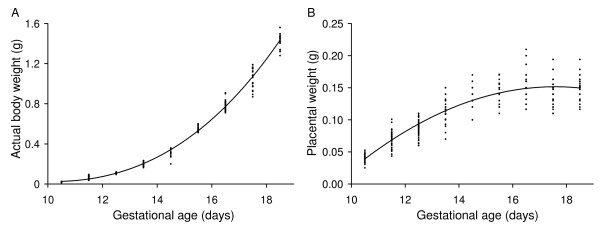
**Embryonic and placental growth quantified using ex vivo weight**. Measured (A) embryo and (B) placental weights are shown as a function of gestational age. Each point shows the result obtained in one conceptus. Lines were generated using the regression equations shown in Table 1.

Placental diameter and placental thickness were found to increase non-linearly with gestational age (Table 1, Fig. 7). Both measures of placental size increased progressively from E10.5 to ~E14.5 then remained constant to term (Fig. 7). A growth plateau in late gestation is in accord with the plateau observed in placental weight measurements (Fig. 8B) and contrasts with continued late-gestational increases in fetal body weight (Fig. 8A) and umbilical blood flow velocity [33]. The late-gestational plateau in placental growth corresponds to a maturational phase of placental development in which vascularity increases and the thickness of the materno-fetal interhaemal barrier decreases [34] thereby enhancing placental transfer efficiency.

**Figure 9:**
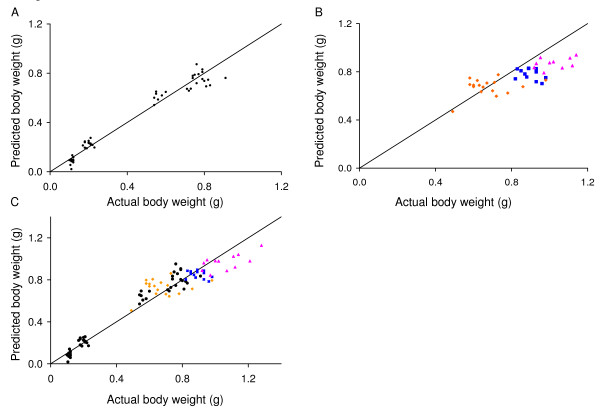
**Correspondence between predicted and measured embryonic body weight**. (A) Embryo weight predicted using the multiple regression equation based on ultrasound measurement of crown-rump length and abdominal circumference in CD-1 mice versus measured body weight for each CD-1 embryo. (B) Equation derived from data obtained in CD-1 mice applied to three other strains of mice (C57Bl/6J (dark blue squares), B6CBAF1 (pink triangles), and hIGFBP1 transgenics (orange diamonds)). (C) Equation derived using data from all four strains is shown applied to all four strains (CD-1 (black circles), C57Bl/6J (dark blue squares), B6CBAF1 (pink triangles), and hIGFBP1 transgenics (orange diamonds)). Each point shows the result obtained in one conceptus. The lines show the line of identity (where y = x).

In human pregnancy, ultrasound parameters are routinely used to estimate fetal gestational age and body weight. Thus, we used regression analysis to generate equations to predict gestational age from individual ultrasound parameters (Table 3). We used CD-1 mice, an out-bred strain often used in reproductive research because it is a reliable and prolific breeder. These equations may be useful in future studies on CD-1 mice to estimate embryonic age when the plug date is unknown. We also used the parameters of crown-rump length, abdominal circumference, and biparietal diameter alone and in combination to generate equations to predict embryonic body weight (Table 4). We found that crown-rump length and abdominal circumference provided a good prediction of embryonic body weight (Fig. 9A) and that there was no significant improvement achieved with the inclusion of biparietal diameter (not shown). We also evaluated the ability of this equation to predict embryonic weights in three other strains of mice with embryos of discrepant size. We used C57Bl/6J and B6CBAF1 mice because they are common background strains for genetically-altered mice, and a hIGFBP1 transgenic model [35] as an example of a genetically-altered mouse model with intrauterine growth restriction. The prediction equation BW = -0.757 + 0.0453 (CRL) + 0.0334 (AC) derived from CD-1 data was used to predict embryonic weights at E17.5 in C57Bl/6J, B6CBAF1, and hIGFBP1 transgenic mice (Fig. 9B). The fit tended to diverge from predicted for embryo weights >0.8 g (Fig. 9B). This may be because these weights are largely in the extrapolated range of the equation or, alternatively, because the equation overestimates these weights due to strain differences. Nevertheless, the mean absolute discrepancy for C57Bl/6J, B6CBAF1, and hIGFBP1 transgenic embryos was 12, 16, and 13% respectively (Fig. 9B) which was similar to the value of 14% calculated for CD-1 mice (E12.5 – E16.5; Fig. 9A). A body weight prediction equation using data from all four strains was also derived (Table 5). Again, crown-rump length and abdominal circumference were found to be the best predictors, with no significant improvement afforded by the inclusion of biparietal diameter. We evaluated the fit of this equation (BW = -0.858 + 0.0659(CRL) + 0.0257(AC)) to the measured body weights of the four strains (Fig. 9C). The mean absolute discrepancy using this equation was 15% and thus was similar to that obtained using the equation derived from CD-1 data alone.

**Table 5:** Prediction equations for body weight (in g) from measured variables (in mm) using data from four strains

From crown-rump length (CRL):
BW = -0.778+ 0.0966(CRL)
R^2 ^= 0.906

From biparietal diameter (BPD):
BW = 12.21 - 7.96(BPD) + 1.67(BPD)^2 ^- 0.110(BPD)^3^
R^2 ^= 0.844

From abdominal circumference (AC):
BW = -0.08 - 0.018(AC) + 0.00247(AC)^2^
R^2 ^= 0.879

From crown-rump length (CRL) and abdominal circumference (AC):
BW = -0.858 + 0.0659(CRL) + 0.0257(AC)
R^2 ^= 0.918

BW, body weight

The use of 40 MHz ultrasound for phenotypic analysis of the conceptus also has important limitations including the skill required and the relatively high cost of the equipment. In addition, it is often difficult to achieve the optimal view for morphometric measurements and this is an important source of measurement error. Depending on the number of embryos and their location, some live embryos may not be visible (~10% in our experience [20]) and some may not be in an appropriate orientation for accurate measurement. There is also the possibility that bioeffects associated with anesthesia and/or ultrasound could affect subsequent development of the conceptus. 40 MHz ultrasound under isoflurane anesthesia during organogenesis (E8.5 or E10.5) had no significant effect on birth weight and minimal effects on postnatal growth [36]. However, fetal ultrasound [37] and embryonic exposure to isoflurane [38] can affect biological outcomes so appropriate controls are necessary.

## Conclusion

Embryonic and placental growth parameters were quantified using 40 MHz ultrasound generating normal growth curves over parameter-specific gestational intervals. Parameters tested exhibited no systematic errors relative to ex vivo measurements by light microscopy, and embryonic body weights estimated using equations derived from CD-1 mice were similarly accurate in three other mouse strains. We found that in vivo quantification of placental size is adequate to detect the normal cessation of placental growth that occurs at ~E14.5. The capacity to quantify placental growth in vivo is important given the crucial role of the placenta in supporting embryonic growth, and our limited understanding of placental growth control. Thus, micro-ultrasound provides a feasible means for obtaining detailed information on prenatal embryonic and placental growth characteristics in genetically-altered and/or environmentally-challenged mouse models, and may also prove useful for estimating gestational age and/or embryonic body weight in utero.

## Competing interests

SLA was a member of the Scientific Advisory Board of VisualSonics from 2003 to 2006 but otherwise has no financial interests in the company.

## Authors' contributions

JM conceived the study, JM and SLA participated in study design, JM and JCS performed ultrasound imaging, DQ participated in breeding and study coordination, SM performed statistical analysis and prepared graphs and tables, and JM and SLA drafted the manuscript. All authors read and approved the final manuscript.
